# Hyperglycemia-induced Occipital Lobe Seizures

**DOI:** 10.1210/jcemcr/luaf223

**Published:** 2025-09-30

**Authors:** Emily Resisi, John Zadran, Daniel Kurtz, Calvin Yu, Lachonne Anderson

**Affiliations:** Department of Medicine, New York Institute of Technology College of Osteopathic Medicine, Glen Head, NY 11545, USA; Icahn School of Medicine at Mount Sinai, Department of Medicine, Mount Sinai South Nassau, Oceanside, NY 11572, USA; Icahn School of Medicine at Mount Sinai, Department of Medicine, Mount Sinai South Nassau, Oceanside, NY 11572, USA; Icahn School of Medicine at Mount Sinai, Department of Medicine, Mount Sinai South Nassau, Oceanside, NY 11572, USA; Icahn School of Medicine at Mount Sinai, Department of Medicine, Mount Sinai South Nassau, Oceanside, NY 11572, USA

**Keywords:** occipital lobe seizures, vision loss, type 2 diabetes, subcortical T2 hypointensity

## Abstract

Hyperosmolar hyperglycemic state (HHS) and nonketotic hyperglycemia (NKH) are recognized complications of type 2 diabetes mellitus that can cause seizures. Occipital lobe seizures with isolated visual symptoms are rare and underrecognized. We present the case of a 70-year-old male with uncontrolled type 2 diabetes who experienced sudden onset of positive visual phenomena and a right inferior visual field defect. The patient had a hemoglobin A1c > 14% (∼130 mmol/mol) (reference range: < 6.5%; < 48 mmol/mol), no ketones, a normal anion gap, and a normal serum osmolality. Brain magnetic resonance imaging without contrast revealed subtle subcortical T2 hypointensity in the left occipital pole and an electroencephalogram showed a left occipital seizure focus. The patient did not meet HHS criteria, showing NKH alone may trigger occipital seizures. Following levetiracetam and insulin therapy, the patient's symptoms improved within an hour. At 7-month follow-up, he reported no recurrence. This case highlights the importance of early recognition of hyperglycemia-induced occipital seizures and suggests that subcortical T2 hypointensity may serve as a valuable diagnostic clue.

## Introduction

Hyperosmolar hyperglycemic state (HHS) is a life-threatening complication of type 2 diabetes mellitus, characterized by extreme hyperglycemia of ≥600 mg/dL (reference range: 70-100 mg/dL; 3.89-5.55 mmol/L), serum osmolality >320 mOsm/kg (reference range: 278-305 mOsm/kg), dehydration, and altered mental status [1]. Nonketotic hyperglycemia (NKH), a related but less severe condition, typically presents with marked hyperglycemia, a normal or mildly elevated serum osmolality, and absence of ketones or acidosis. In contrast to diabetic ketoacidosis, which reflects absolute or relative insulin deficiency and can occur in either type 1 or type 2 diabetes, patients with type 2 diabetes retain some insulin activity, preventing ketone production. The mortality rate of HHS can be as high as 20%, which is much higher than in diabetic ketoacidosis [1]. HHS can be triggered by infections, dehydration, medications (most commonly glucocorticoids, thiazides, and beta-blockers), or cardiovascular events [1].

Seizures in the setting of severe hyperglycemia are relatively uncommon, with most reports drawn from isolated cases or small series [1-3]. Occipital lobe seizures are rare, with only scattered case reports. They may cause transient visual disturbances without motor symptoms. Understanding their presentation is critical for early intervention, as a missed diagnosis can delay treatment and recovery. We present a unique case of NKH-induced occipital seizures in a patient without HHS, and we discuss its clinical, radiologic, and electrographic features.

## Case Presentation

A 70-year-old male with a history of type 2 diabetes mellitus, hypertension, and hyperlipidemia presented with abrupt right inferior visual field loss and positive visual phenomena described as “blooming flowers” that persisted even with his eyes closed. These symptoms began 3 days before presentation while lifting a heavy object. He also reported dull, generalized headaches for the last 3 days. There was no history of similar episodes, migraines, seizures, or epilepsy.

He was diagnosed with diabetes 1 year prior by his primary care physician and was prescribed a combination glyburide–metformin 5 mg/500 mg daily. He admitted to poor adherence, citing forgetfulness and a belief that diabetes was not serious. He was never followed by an endocrinologist or placed on insulin. He denied a family history of diabetes or seizures.

His diet was high in carbohydrates, consisting of rice, bread, and cakes, with 5 small meals daily. He reported adequate fluid intake with multiple glasses of water per day. His weight was stable around 263 pounds (119.3 kg), his height was 5′10″ (1.78 m), and his body mass index was 37.6 kg/m². The physical exam revealed only a right inferior visual field defect. No signs of dehydration were noted, with normal skin turgor, moist mucosae, and pink conjunctiva.

An ophthalmologist confirmed a normal retinal and optic disc exam. Given the persistence of visual symptoms, he was admitted for neurologic evaluation.

## Diagnostic Assessment

On admission, fingerstick blood glucose was 411 mg/dL (22.8 mmol/L) (reference range: 70-100 mg/dL; 3.89-5.55 mmol/L), hemoglobin A1c was >14% (∼130 mmol/mol) (reference range: < 6.5%; < 48 mmol/mol). Serum osmolality was 295 mOsm/kg (reference range: 278-305 mOsm/kg). Serum sodium was 135 mmol/L (reference range: 135-145 mmol/L) and blood urea nitrogen was 15 mg/dL (5.36 mmol/L) (reference range: 7-20 mg/dL; 2.5-7.1 mmol/L), consistent with normal osmolality. Urinalysis and serum studies were negative for ketones. Beta-hydroxybutyrate was 0.19 mmol/L (reference range: < 0.30 mmol/L). Electrolytes were within the normal range except for a mildly low total calcium of 8.2 mg/dL (2.05 mmol/L) (reference range: 8.5-10.5 mg/dL; 2.1-2.6 mmol/L), which increased to 8.7 mg/dL on day 2. Ionized calcium, total protein, albumin, and PTH levels were not obtained. No clinical signs of hypocalcemia were observed.

Vital signs on admission were within normal limits. The patient was afebrile with a body temperature of 97.2°F (36.2 °C) (reference range: 97.0-99.0°F; 36.1-37.2 °C). C-reactive protein was 5.9 mg/L (reference range: < 5.1 mg/L), erythrocyte sedimentation rate was 93 mm/hr (reference range: 0-15 mm/hr). White blood cell count was 8.8 × 10⁹/L (reference range: 4.5-10.3 × 10⁹/L). Procalcitonin, D-dimer, and troponin were not measured. The elevated erythrocyte sedimentation rate and C-reactive protein were considered reactive in the setting of uncontrolled diabetes.

**Figure 1.**
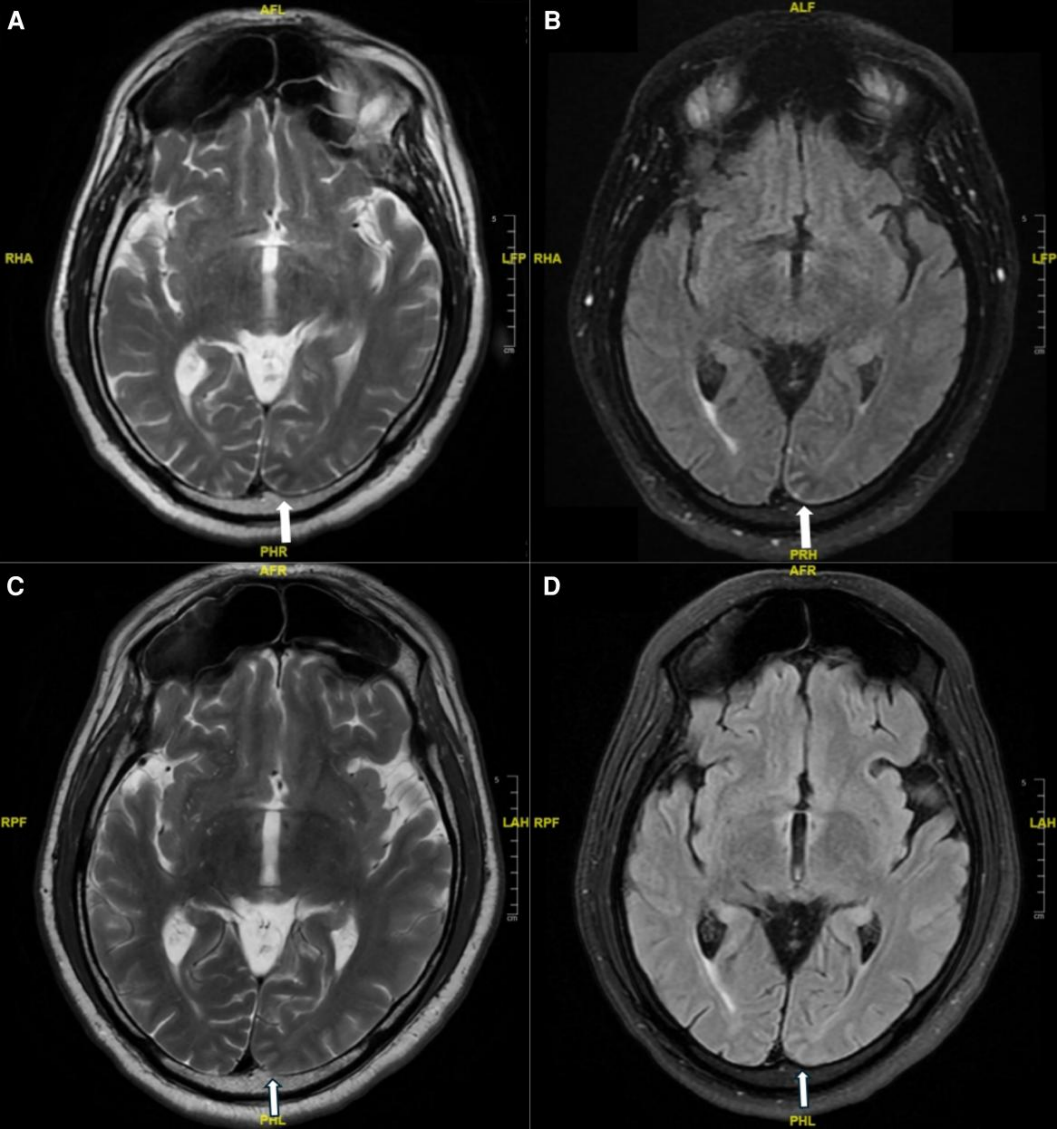
Brain magnetic resonance imaging without contrast demonstrating a subcortical T_2_ hypointensity in the left occipital pole (arrows) at initial presentation (panels A and B) with near-complete resolution on repeat imaging 11 weeks later (panels C and D).

Magnetic resonance imaging (MRI) brain without contrast on hospital day 1 showed subtle subcortical T2/fluid-attenuated inversion recovery hypointensity in the left occipital pole (Fig. 1). Approximately 36 hours later, a follow-up brain MRI with IV contrast showed no enhancement or structural abnormalities. Computed tomography (CT) of the head, CT perfusion imaging of the brain, CT angiography of the circle of Willis, and MRI of the orbits were unremarkable.

**Figure 2.**
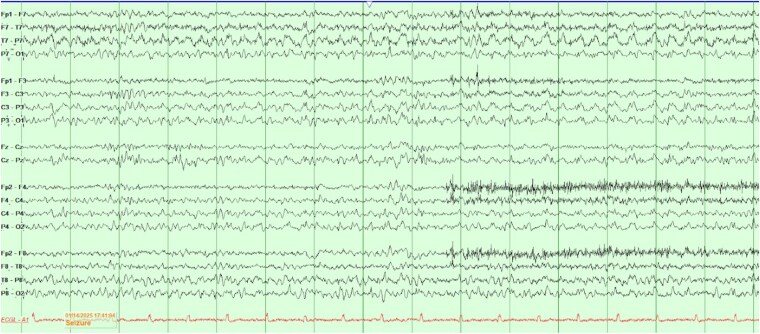
Routine electroencephalogram demonstrating 1 electrographic seizure in the left occipital region, runs of left occipital lateralized periodic discharges, and focal epileptiform discharges involving the left occipital region (O1/P7).

On hospital day 2, an electroencephalogram (EEG) revealed 1 electrographic seizure in the left occipital region (O1/P7), with lateralized periodic discharges (Fig. 2). These findings were concordant with MRI abnormalities and the patient's visual symptoms. Prolonged EEG monitoring was performed over 4 hours, with no further seizures or discharges observed. The EEG study was discontinued due to discomfort.

## Treatment

He was initially treated under a stroke protocol and was administered a loading dose of aspirin 325 mg, then aspirin 81 mg daily, atorvastatin 80 mg, and lisinopril 10 mg daily. Upon EEG confirmation of seizures, a 3 g IV loading dose of levetiracetam was administered, followed by 500 mg twice daily. Insulin therapy was initiated with glargine 15 units nightly and lispro 5 units 3 times daily. Glyburide–metformin was discontinued.

He received two 1-L saline boluses, then 0.9% NaCl at 125 mL/hr for 3.5 days. Fluids were tapered as glucose levels stabilized. Clonazepam 0.5 mg was prescribed as a rescue per local protocol for seizures lasting ≥5 minutes.

Lifestyle counseling, diabetes education, and follow-up planning with endocrinology and neurology were provided prior to discharge.

## Outcome and Follow-up

The patient noted partial resolution of visual symptoms within 1 hour of receiving levetiracetam. Blood glucose decreased to 189 mg/dL upon discharge. At 1-week follow-up, visual phenomena had improved. By week 2, they had resolved completely. At 11 weeks, a follow-up brain MRI showed near-complete resolution of the T2/fluid-attenuated inversion recovery hypointensities, and 48-hour ambulatory EEG was unremarkable.

At 5 months posthospitalization, hemoglobin A1c was 8.1%, and he admitted inconsistent medication use. At 7-month follow-up, the patient reported sustained resolution of seizures. He had lost 50 lb (22.7 kg), attributed to regular meals with increased fiber and reduced carbohydrates. He reported occasional morning headaches without aura. No new neurological deficits were observed.

## Discussion

Seizures are rare but documented complications of hyperglycemic crises. The pathophysiology remains uncertain, but proposed mechanisms include decreased gamma-aminobutyric acid due to absent ketones, adenosine triphosphate depletion within neurons, and osmotic stress resulting from hyperglycemia-induced cellular dehydration [3-5]. Occipital seizures often present with positive visual symptoms such as hallucinations, photopsia, or transient blindness [4]. This case supports that prolonged hyperglycemia can provoke cortical hyperexcitability in the occipital lobes.

Although this case reinforces the association between NKH and occipital lobe seizures, it also raises the question of whether hyperglycemia alone fully accounts for the patient's visual symptoms. Hyperosmolar states can lead to cortical dysfunction through shifts in fluid and electrolytes, yet phenomena such as visual hallucinations, scotomas, and transient cortical blindness are not always explained by glycemic changes alone [1, 5]. Such symptoms may reflect seizures, ischemia, or subtle metabolic changes.

Neuroimaging often appears unremarkable in these settings [3, 5]. However, an emerging trend in the literature points to subcortical T2 hypointensities as a subtle but potentially meaningful radiologic marker. Our patient's MRI demonstrated this finding, and with the absence of HHS, this suggests that persistent or severe hyperglycemia alone may be sufficient to provoke cortical excitability and seizures. Similar observations have been described in reports by Sasaki et al, Alakkas et al, and Lee et al [6-8].

Table 1 outlines several published reports involving patients with hyperglycemia-related visual disturbances. These include both seizure-associated and transient vision-related phenomena. Collectively, these cases support the need to consider seizure activity in patients with poorly controlled diabetes presenting with acute visual symptoms. Subcortical T2 signal changes on MRI are subtle but may aid in identifying patients who would benefit from EEG evaluation and early treatment.

**Table 1.** Selected case reports and small series of visual/seizure-related manifestations in hyperglycemia

Reference	Patient demographics	Glycemic status	Symptoms	Imaging/EEG findings
Putta et al [2]	67-year-old male	NKH	Visual hallucinations, seizures	Occipital subcortical T₂ hypointensity
Sasaki et al [6]	72-year-old male	NKH	Right visual field defect, hallucinations	Left occipital subcortical T₂ and T₂* hypointensity; occipital seizures on EEG
Alakkas et al [7]	70-year-old male	Hyperglycemia (no HHS criteria)	Occipital seizures with visual symptoms	Subcortical T₂ hypointensity
Lee et al [8]	65-year-old female	NKH	Photopsia, transient visual aura	Occipital spikes on EEG
Engez et al [9]	56-year-old male	NKH	Visual phenomena (flashing lights), persistent homonymous hemianopia	Subcortical T₂/FLAIR hypointensity in occipital lobes; occipital epileptiform discharges
Baltyde et al [10]	Retrospective series: 18/228 diabetic patients with seizures due to NKH	NKH	Transient focal motor or generalized seizures, some with visual symptoms	Imaging often unremarkable

Abbreviations: EEG, electroencephalogram; FLAIR, fluid-attenuated inversion recovery; HHS, hyperosmolar hyperglycemic state; MRI, magnetic resonance imaging; NKH, nonketotic hyperglycemia; T₂*, apparent transverse relaxation time (gradient-echo).

Numerous reports have documented visual and neurologic symptoms in patients with poorly controlled diabetes, although the presentations can vary widely. Some individuals have been noted to experience vivid hallucinations or even involuntary movements, such as brief arm jerks or twitches [4-6]. A case by Putta et al described a patient with occipital seizures and subcortical T2 hypointensity on MRI, similar to our findings [2]. HHS has also been linked to movement disorders like hemichorea and hemiballismus, as well as more diffuse symptoms like global aphasia [11].

Taken together, these cases emphasize the importance of maintaining a broad differential in diabetic patients who present with new-onset visual disturbances. While diabetic retinopathy is a common concern in patients with visual changes, normal retinal findings should prompt further evaluation. In those with poorly controlled diabetes, brain imaging is key to identifying potential seizure activity related to hyperglycemia.

This case also serves as a reminder of the need to routinely reassess diabetes medications in older adults. The patient had been taking glyburide, a sulfonylurea discouraged in older adults due to its prolonged half-life and hypoglycemia risk [12, 13]. While he did not experience hypoglycemia during this admission, therapy was adjusted to reduce future metabolic risk.

There are limitations worth noting in this case. Although the diagnosis was supported by EEG and imaging, continuous EEG monitoring was limited to a short duration, potentially missing further seizure activity. In addition, metabolic markers such as ionized calcium and PTH were not obtained, which may have provided more insight into cortical excitability. These factors limit comprehensive assessment but do not diminish the clinical relevance of this presentation.

## Learning Points

- Occipital seizures may present solely with visual symptoms in poorly controlled type 2 diabetes, even without HHS.
- Subcortical T2 hypointensity on MRI can be a transient finding that resolves with improved glucose control.
- New-onset visual symptoms in diabetics warrant early EEG and MRI to detect possible seizure activity.
- Management should emphasize glycemic control, patient education, adherence support, and avoidance of long-acting sulfonylureas in older adults.

## Contributors

E.R. and J.Z. contributed equally to this work, were involved in the clinical diagnosis and management of the patient, performed the literature review, and drafted the manuscript. L.A. and C.Y. assisted with patient care, data collection, and critical revision of the manuscript for intellectual content. D.K. contributed to clinical management, literature review, and manuscript editing. J.Z. coordinated the submission process. All authors reviewed and approved the final version of the manuscript.

## Data Availability

Data sharing is not applicable to this article as no datasets were generated or analyzed during the current study.
